# Circulating syndecan-1 and glypican-4 predict 12-month survival in metastatic colorectal cancer patients

**DOI:** 10.3389/fonc.2022.1045995

**Published:** 2022-10-24

**Authors:** Axel Muendlein, Luciano Severgnini, Thomas Decker, Christine Heinzle, Andreas Leiherer, Kathrin Geiger, Heinz Drexel, Thomas Winder, Patrick Reimann, Frank Mayer, Christoph Nonnenbroich, Tobias Dechow

**Affiliations:** ^1^ Vorarlberg Institute for Vascular Investigation and Treatment, Molecular Biology Laboratory, Dornbirn, Austria; ^2^ Department of Haematology and Oncology, Academic Teaching Hospital Feldkirch, Feldkirch, Austria; ^3^ Private University of the Principality of Liechtenstein, Triesen, Liechtenstein; ^4^ Onkologie Ravensburg, Ravensburg, Germany; ^5^ Medical Central Laboratories, Feldkirch, Austria; ^6^ Department of Internal Medicine, Academic Teaching Hospital Bregenz, Bregenz, Austria; ^7^ Praxis und Tagesklinik Prof. Dr. Oettle Helmut Prof. Mayer Frank, Friedrichshafen, Germany

**Keywords:** colorectal cancer, proteoglycans, shedding, syndecan-1 (SDC1), glypican-4, biomarker, prognosis

## Abstract

Cell surface syndecans and glypicans play important roles in the development and prognosis of colorectal cancer (CRC). Their soluble forms from proteoglycan shedding can be detected in blood and have been proposed as new prognostic biomarkers in several cancer entities. However, studies on circulating syndecan-1 (SDC1) and glypican-4 (GPC4) in CRC are limited. We, therefore, evaluated the impact of plasma SDC1 and GPC4 on the prognosis of metastatic (m)CRC patients. The present study included 93 patients with mCRC. The endpoints were progression-free survival (PFS) and overall survival (OS) at 12 months. SDC1 and GPC4 levels were measured in plasma using enzyme-linked immunosorbent assays. Plasma levels of SDC1 and GPC4 were significantly correlated. Significant correlations of these two markers were also found with carcinoembryonic antigen (CEA). Kaplan-Meier curve analyses indicated that PFS and OS probabilities significantly decreased with increasing levels of SDC1 and GPC4, respectively. Multivariable Cox regression analyses showed that both markers were significantly associated with PFS and OS independently from clinicopathological characteristics including CEA. Respective adjusted hazard ratios (HR) together with corresponding 95% confidence intervals for one standard deviation change of SDC1 were 1.32 [1.02-1.84] for PFS and 1.48 [1.01-2.15] for OS. Adjusted HRs [95% confidence intervals] of GPC4 were 1.42 [1.07-1.89] for PFS and 2.40 [1.51-3.81] for OS. Results from area under the receiver operating characteristic curve analyses suggest that GPC4 and SDC1 add additional prognostic values to CEA for OS. In conclusion, we showed significant associations of circulating SDC1 and GPC4 with poor survival of mCRC patients.

## Introduction

Colorectal cancer (CRC) is the third most prevalent cancer diagnosis in the world (1). At least half of the patients with diagnosed CRC will develop metastatic CRC (mCRC) (2). Despite recent advances in systemic treatment options and surgical management strategies (3, 4) prognosis of mCRC remains grim, with a relative 5‐year survival rate of only 14% (5).

Several studies found a central and significant contributory role of members of the heparan sulphate proteoglycan (HSPG) family in the development and prognosis of cancer, including CRC (6, 7). HSPGs are mainly present in the cell membrane and extracellular matrix, where they may interact with cytokines, chemokines, and growth factors playing active roles in cell adhesion, migration, proliferation, and signaling pathways. Syndecans, which have a transmembrane domain in their core proteins, and glycosylphosphatidylinositol-anchored glypicans are the two main subfamilies of membrane HSPGs (8).

Syndecan-1 (SDC1, alias CD138) is the major cell-surface proteoglycan in endothelial cells (9, 10) and is involved in the remodeling and angiogenesis of CRC tissue (11). Loss of SDC1 expression in CRC cells has been associated with metastatic potential and shorter survival in several (11–13) but not all studies (14). SDC1 can be released from the cell surface into the bloodstream after proteoglycan shedding, mainly induced by matrix metalloproteinases and other proteins (15). Activated shedding of membrane SDC1 and other HSPGs is mainly observed under various pathogenic conditions, including cancer (6, 15). However, only a few studies have investigated the association of circulating SDC1 with the prognosis of CRC linking serum SDC1 with chemotherapy resistance and survival in mCRC patients (16, 17).

Glypican-4 (GPC4) is another cell-surface proteoglycan involved in regulating several oncogenic pathways including Wnt, bone morphogenic protein, fibroblast growth factor 2, and hepatocyte growth factor (18–20). However, studies on GPC4 in cancer are rather limited. GPC4 expression has been associated with oxaliplatin resistance in ovarian carcinoma cells (21) and 5-fluorouracil resistance in pancreatic cancer cells (22). Shedded GPC4 can be detected in blood also and has been linked with insulin resistance (23) as well as with several metabolic disorders (23–30). However, the association between circulating GPC4 and the prognosis of patients with mCRC or other types of cancer is unknown so far.

In the present study, we therefore prospectively analyzed the impact of circulating SDC1 and GPC4 on the survival of mCRC patients and evaluated their prognostic values as new biomarkers for mCRC.

## Materials and methods

### Study subjects

From October 2017 through June 2020, patients with mCRC were assessed for eligibility at the ‘Oncology Study Center Ravensburg’ (Ravensburg, Germany) at the ‘Department of Hematology, Oncology, Gastroenterology and Infectiology’ of the Academic Teaching Hospital Feldkirch (Feldkirch, Austria), and at a private medical practice in Friedrichshafen, Germany (‘Gemeinschaftspraxis Oettle und Mayer’). Eligible patients were aged ≥18 years and diagnosed with mCRC starting first line or second line of therapy. All patients had to give written informed consent for participation in the present study. Baseline clinicopathological parameters were obtained from medical records, including age, sex, tumor histopathology, RAS mutation status, metastatic status, serum carcinoembryonic antigen (CEA) levels, and scheduled medical treatments. Patients were followed up until disease progression, death or end of the observational period on June 30th, 2021. The endpoints were progression-free survival (PFS; defined as the time from baseline to disease progression or death from any cause) and overall survival (OS) at 12 months. Only patients with available clinical and laboratory data and complete follow-up were included in the present study. The study protocol was approved by the Ethics Commission of the State Chamber of Medicine of Baden-Württemberg (Germany) and by the Ethics Committee of the State of Vorarlberg (Austria) and is following the 1964 Helsinki declaration and its later amendments or comparable ethical standards.

### Laboratory measurements

At baseline, blood samples were collected, and plasma samples were stored at -80°C until used for the analysis of SDC1 and GPC4, respectively. Circulating SDC1 and GPC4 levels were determined *via* commercially available enzyme-linked immunosorbent assays (ELISA; Thermo Fisher Scientific, Waltham, MA USA; article number: EHSDC1 and Cloude-Clone; Houston, Texas; article number: SEA998Hu, respectively) following the manuals of the manufacturers.

### Statistical analyses

Differences in baseline characteristics according to tertiles of SDC1 and GPC4 were tested for statistical significance with Chi-squared tests for trend for categorical and Jonckheere Terpstra tests for continuous variables, respectively. Continuous variables are given as median and interquartile range (defined as the range from the 25^th^ to the 75^th^ percentile). Normal distribution was assessed using Kolmogorov-Smirnov indicating that SDC1 and GPC4 values were not normally distributed (each p-value <0.001). Non-normal distribution of plasma SDC1 and GPC4, respectively, was visually verified using a quantile-quantile plot as displayed in **Supplementary Figure 1**. Correlation analyses were performed by calculating non-parametric Spearman rank correlation coefficients. Differences between SDC1 and GPC4 levels and categorical variables were tested for statistical significance using the Mann–Whitney–U test. Survival curves were generated using the Kaplan-Meier method and compared using log-rank-Mantel-Cox tests. Hazard ratios (HRs) and 95% confidence intervals (CIs) of the HRs were derived from univariable and multivariable Cox proportional hazards models. Z-transformation was applied before logistic regression analysis. In addition, area under the receiver operating characteristic curve (ROC-AUC) analyses were performed and the statistical significance of the difference between AUCs was tested with the method of DeLong (31). To examine the prognostic values of SDC1 and GPC4 as prognostic biomarkers for mCRC, binary logistic regression models were fitted with PFS and OS, respectively, as the dependent variable. A basic model comprising serum CEA, which represents one of the most extensively used prognostic biomarkers in CRC (32), as the independent variable was compared with further models including SDC1 and/or GPC4 as additional markers. Statistical analyses were performed with SPSS 28.0.0 (SPSS, Inc., Chicago, IL) and GraphPad Prism 8 (GraphPad Software, San Diego, CA).

## Results

### Patients’ characteristics

Stored plasma samples for SDC1 and GPC4 analysis were available from 93 patients with complete follow-up data, which were included in the present study. The clinicopathological characteristics of the study cohort are shown in **Table 1**. Patients’ characteristics stratified by tertiles of SDC1 and GPC4 are given in **Supplementary Tables 1** and **2**, respectively. Age, sex, primary tumor localization, number of metastatic sites, and kind of scheduled therapy did not differ significantly between tertiles of SDC1 or GPC4. GPC4 levels increased with increasing SDC1 tertiles and vice versa (each *P*-value < 0.001). Spearman’s correlation analysis using SDC1 and GPC4 as continuous variables confirmed the highly significant correlation between these two parameters (rho = 0.521; *P* < 0.001). In addition, CEA levels were significantly associated with tertiles of SDC1 and GPC4 (*P* < 0.001 and *P* = 0.005, respectively). Respective Spearman’s correlation coefficient values were rho = 0.484; *P* < 0.001 and rho = 0.351; *P* < 0.001 for soluble SDC1 and GPC4, respectively, using continuous variables. Scatter plots visualizing the correlations between SDC1, GPC4, and CEA are displayed in **Supplementary Figure 2**.

**Table 1:** Basic clinical and clinicopathological characteristics of the total cohort.

	Total cohort
Age, years	65.8 [56.5-73.5]
Sex	
Male, N (%)	61 (65.6)
Female, N (%)	32 (34.4)
Primary tumor site	
Colon, N (%)	62 (66.7)
Rectum, N (%)	31 (33.3)
Primary tumor sidedness^†^	
Left, N(%)	65 (73.0)
Right, N(%)	24 (27.0)
RAS mutation status^††^	
Wild-type, N (%)	45 (53.6)
Mutated, N (%)	39 (46.4)
Number of metastatic sites^††^	
1, N (%)	46 (54.8)
2, N (%)	26 (31.0)
≥3, N (%)	12 (14.3)
Scheduled therapy line	
1^st^, N (%)	84 (90.3)
2^nd^, N (%)	9 (9.7)
Scheduled chemotherapy	
FOLFIRI, N (%)	55 (59.1)
FOLFOX, N (%)	14 (15.1)
Other chemotherapy, N (%)	24 (25.8)
Scheduled targeted therapy^†††^	
No targeted therapy, N (%)	16 (17.6)
VEGF-inhibitor therapy, N (%)	47 (51.6)
EGFR-inhibitor therapy, N (%)	28 (30.8)
Laboratory parameters	
CEA, ng/mL	24.0 [5.8-100]
Syndecan-1, ng/mL	4.31 [2.81-6.07]
Glypican-4, ng/mL	5.61 [4.57-8.72]

Missing samples: † n=4; †† n=9; ††† n=2. Continuous variables are given as median and interquartile range (defined as the range from the 25^th^ percentile to the 75^th^ percentile).

### Prospective study

**Figure 1:**
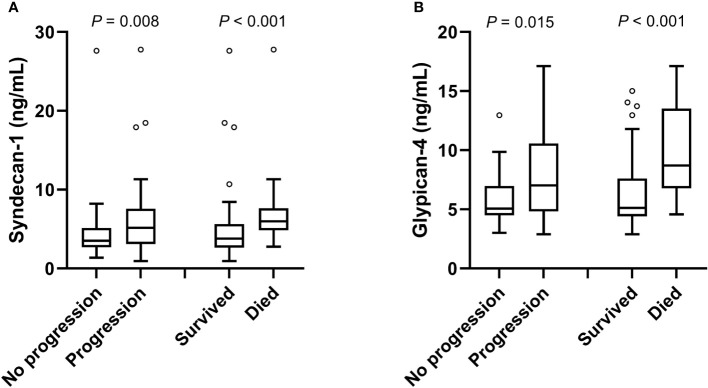
Plasma levels of SDC1 and GPC4 with respect to disease progression and mortality. Plasma levels of **(A)** SDC1 and **(B)** GPC4 with respect to disease progression and mortality are shown as box plots using the Tukey method for plotting the whiskers and outliers. P-values were calculated using the Mann-Whitney U test.

**Figure 2:**
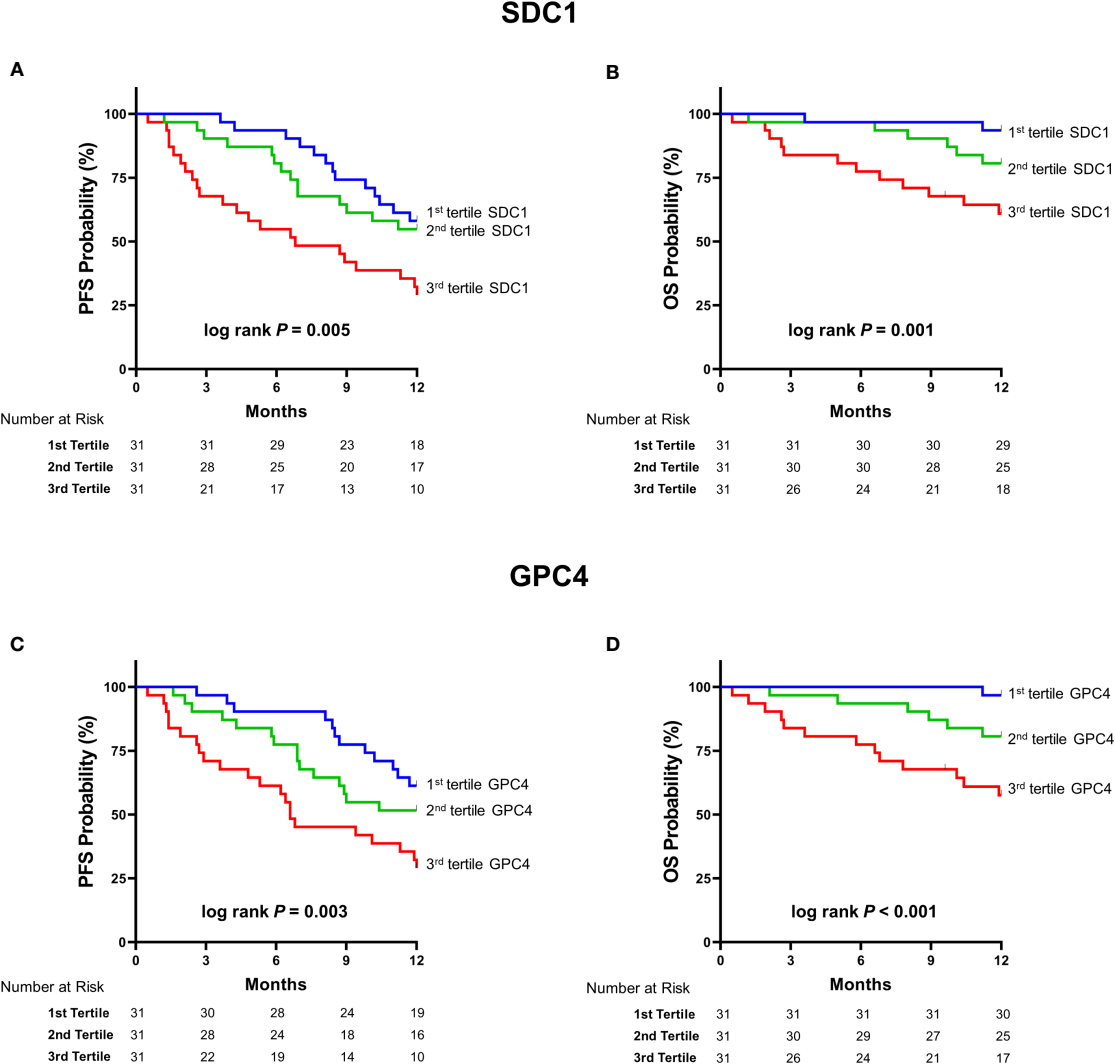
Kaplan Meier estimates of survival according to tertiles of SDC1 and GPC4. Kaplan–Meier curves of **(A)** PFS stratified by tertiles of SDC1 **(B)** OS stratified by tertiles of SDC1 **(C)** PFS stratified by tertiles of GPC4 **(D)** OS stratified by tertiles of OS. P-values were obtained by Log-Rank-Mantel-Cox-tests. SDC1, syndecan-1; GPC4, glypican-4; PFS, progression-free survival; OS, overall survival.

**Figure 3:**
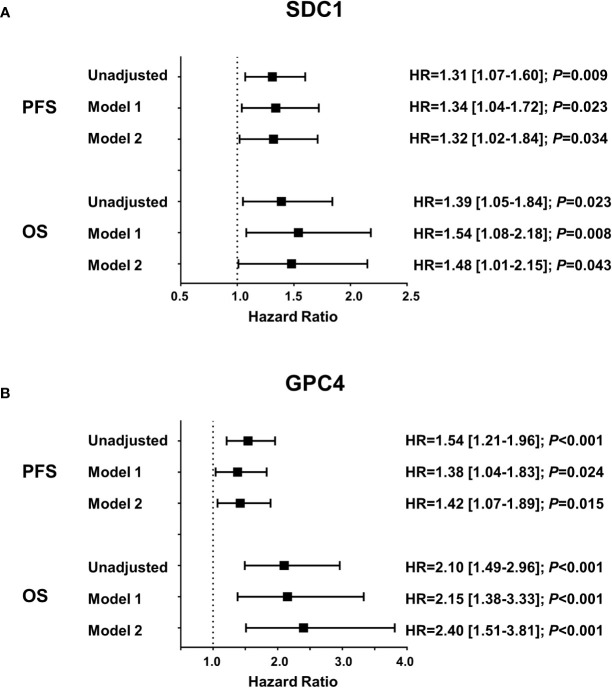
Association of plasma SDC1 and GPC4 with progression free survival and overall survival. Results were obtained from Cox proportional hazards regression analyses and are presented as hazard ratios (HR) and 95% confidence intervals [CI] for one standard deviation change of **(A)** SDC1 and **(B)** GPC4. Model 1 includes age, sex, number of metastatic sites, and scheduled therapy; model 2 additionally includes CEA. PFS, progression-free survival; OS, overall survival; SDC1, syndecan-1; GPC4, glypican-4; CEA, carcinoembryonic antigen.

Circulating SDC1 and GPC4 values were significantly increased in patients showing a progression of the disease (52.7%; n=49) and in patients who died (21.5%; n=20) during the 12-month follow-up period (**Figure 1**). Kaplan-Meier estimates representing the probabilities of PFS and OS stratified by tertiles of SDC1 and GPC4, respectively, are displayed in **Figure 2**. PFS probability significantly decreased from the 1st tertile of plasma SDC1 over the 2^nd^ SDC1 tertile to the 3^rd^ tertile of SDC1. The same was true for OS. Similarly, increasing GPC4 tertiles were significantly associated with decreased probabilities of PFS and OS. The significant associations of SDC1 and GPC4 with survival were confirmed by univariable and multivariable Cox regression analyses using these markers as continuous variables. Respective hazard ratios together with corresponding confidence intervals are shown in **Figure 3**. Plasma SDC1 and GPC4 were associated with PFS and OS independently from clinicopathological characteristics including CEA. In particular, the association between circulating GPC4 and OS remained highly significant in each regression model. Multivariable Cox regression analysis including both SDC1 and GPC4 in the same regression model further showed that the association with OS remained significant for GPC4 but not for SDC1 (HR = 2.15 [1.43-3.22]; *P* < 0.001 and HR = 0.96 [0.65-1.43]; *P* = 0.847, respectively). The same was true for PFS (HR = 1.47 [1.01–1.97]; *P* = 0.010 and HR = 1.08 [0.83–1.41]; *P* = 0.553, respectively).

Area under the receiver operating characteristic curve (ROC-AUC) analyses were used to compare the performance of SDC1 and GPC4 as biomarkers for PFS or OS with CEA. **Table 2** shows the AUCs and the results from DeLong’s test indicating a significant additional prognostic value of SDC1 and/or GPC4 to a model comprising CEA as the independent variable for the 12-month OS. Highest AUC for OS was observed for the two-parameter model comprising GPC4 and CEA. That said, no additional prognostic value of SDC1 and/or GPC4 to CEA was observed for PFS.

**Table 2:** Results from area under the receiver operating characteristic curve analyses.

		AUC (95% CI)	Z for DeLong’s test	*P*-value
PFS	CEA	0.595 [0.479-0.711]		–
	SDC1	0.660 [550-0.771]	1.087	0.277
	GPC4	0.647 [0.534-0.750]	0.753	0.451
	CEA+SDC1	0.656 [0.544-0.769]	1.120	0.263
	CEA+GPC4	0.655 [0.642-0.769]	0.982	0.326
	CEA+SDC1+GPC4	0.654 [0.541-0.768]	0.973	0.331
OS	CEA	0.638 [0.478-0.798]		–
	SDC1	0.748 [0.637-858]	1.476	0.140
	GPC4	0.795 [0.694-0.896]	1.883	0.060
	CEA+SDC1	0.766 [0.651-0.881]	2.114	0.035
	CEA+GPC4	0.811 [0.708-0.914]	2.447	0.014
	CEA+SDC1+GPC4	0.806 [0.697-0.915]	2.383	0.017

AUCs of SDC1, GPC4, a two-parameter model comprising CEA and either SDC1 or GPC4, and a three-parameter model comprising CEA, SDC1 as well as GPC4 were compared to CEA according to DeLong’s test. The two-parameter model and the three-parameter model were built as linear predictor scores after Cox regression. Abbreviations: AUC, area under the curve; CI, confidence interval; CEA, carcinoembryonic antigen; SDC1, syndecan-1; GPC4, glypican-4; PFS, progression-free survival; OS, overall survival.

## Discussion

In the present study, we showed that high plasma levels of SDC1 and GPC4 independently predicted outcomes in patients with mCRC. Studies on GPC4 in cancer are rather limited and a significant association between plasma GPC4 and the outcome of CRC patients has not been described so far. Furthermore, our study is in line with two previous studies demonstrating that circulating SDC1 is significantly linked with the prognosis of CRC (16, 17). In this regard, Wang et al. showed that CRC patients with high SDC1 serum levels showed a poorer disease-free survival (17) and Jary et al. identified soluble SDC1 as an independent prognostic risk factor of OS in mCRC patients (16). In addition, significant associations between circulating SDC1 and the outcome of patients with other tumor entities including lung cancer (33, 34), prostate cancer (35), bladder cancer (36), and multiple myeloma (37) have been reported in the literature.

It has been suggested that shed SDC1 is associated with chemotherapy resistance, probably linking soluble SDC1 with poor survival of cancer patients. Wang et al. demonstrated that CRC patients with high pre-operative SDC1 levels were less responsive to 5-fluorouracil, oxaliplatin, irinotecan, cisplatin, or paclitaxel chemotherapy (17). As a possible mechanism of chemotherapy resistance in CRC, the authors proposed that shed SDC1 may interact with soluble heparin-binding EGF-like growth factor enhancing the activation of EGFR and downstream signaling, which in turn decreases the chemotherapeutical sensitivity of colon cancer cells (38). It has been further shown that the shed of SDC1 is associated with chemotherapy resistance in castration-resistance prostate cancer (39). In addition, *in vitro* studies showed that shed SDC1 may bind VEGF stimulating endothelial invasion and tumor angiogenesis in myeloma cells (40). In fact, activated SDC1 shedding has been associated with increased metastasis in various cancers, indicating that soluble SDC1 represents a major facilitator for malignant cellular invasion (41). Consequently, targeting SDC1 and preventing SDC1 shedding has been proposed as promising approaches to prevent tumor progression; respective putative therapeutic strategies have been summarized by Guo et al. (41). However, it remains unclear if SDC1 shedding probably involved in paracrine communication within the tumor microenvironment may also lead to quantifiable concentrations of the free SDC1 ectodomain in blood.

Our study further demonstrated that plasma levels of SDC1 and GPC4 were significantly correlated in mCRC patients. Interestingly, multivariable Cox regression analyses as well as ROC-AUC analyses indicated that plasma GPC4 even outperformed SDC1 as a prognostic biomarker of mCRC. The close correlation between these two biomarkers is not surprising because simultaneous shedding of different HSPGs is found under inflammatory and other pathological conditions (42). Consequently, common pathophysiological mechanisms may have led to measurable blood levels of SDC1 and GPC4 predicting the course of mCRC patients. However, concerning the limited knowledge of shed GPC4 on cancer prognosis, the molecule should not be neglected and deserves further investigations.

Serum SDC1 has been proposed not only as a prognostic marker in cancer but also in cardiovascular disease, kidney disease, diabetes, and sepsis [as reviewed by Leppeda et al. (43)]. Circulating GPC4 has been previously associated with several disorders linked to insulin resistance including elevated systolic blood pressure (27), non-alcoholic fatty liver disease (26), and kidney disease (30, 44). Recently, we showed that circulating GPC4 is associated with increased overall mortality risk in coronary angiography patients (45) as well as in patients with peripheral artery disease (46). Notably, cardiovascular diseases, hypertension, and diabetes are common comorbidities in CRC patients (47) and may have a large impact on both blood levels of shed proteoglycans and survival. Therefore, circulating SDC1 and GPC4 might be markers of general organ dysfunction rather than specific tumor markers. In CRC, CEA represents the most extensively used biomarker to monitor disease progression and to estimate CRC patients’ prognosis (32). Nevertheless, there is a growing consensus that using alternative biomarkers may further improve the assessment of mCRC prognosis (48–50). For this reason, the determination of the tumor marker CEA together with markers of organ dysfunction such as SDC1 or GPC4 may appear advantageous in estimating the prognosis of CRC since they can provide complementary information. In fact, our study’s ROC-AUC analyses suggest that circulating SDC1 and/or GPC4 add additional prognostic value to CEA concerning OS.

Our study has several limitations. By design, our study population was composed of consecutively recruited mCRC patients; our results, therefore, are not necessarily applicable to other cancer stages or cancer types. Due to the consecutive study design, included patients were not uniformly treated and the limited sample size does not allow a detailed subgroup analysis based on individual therapy regimes. However, our study population covers a large proportion of mCRC patients seen in clinical practice providing real-world data linking the impact of circulating HSPGs with the outcome of mCRC. The observational design of our study does not allow definite conclusions regarding causal relationships between investigated biomarkers and study outcomes. The molecular backgrounds behind our findings are needed to be addressed in further studies.

In conclusion, we showed significant associations of circulating SDC1 and GPC4 with poor survival of mCRC patients. Future research appears worthwhile to further evaluate the value of circulating proteoglycans as predictors of colorectal cancer.

## Data availability statement

The original contributions presented in the study are included in the article/**Supplementary Material**. Further inquiries can be directed to the corresponding author.

## Ethics statement

The studies involving human participants were reviewed and approved by Ethics Commission of the State Chamber of Medicine of Baden-Württemberg (Germany) and the Ethic Committee of the State of Vorarlberg (Austria). The patients/participants provided their written informed consent to participate in this study.

## Author contributions

AM designed the study, obtained funding, interpretated data, and wrote the manuscript. LS recruited patients, collected and interpretated data, and revised the manuscript for important intellectual content. ThD recruited patients, collected and interpretated data, and critically revised the manuscript for important intellectual content. CH performed laboratory analyses, interpretated data, and critically revised the manuscript for important intellectual content. AL performed statistical analyses and critically revised the manuscript. KG performed laboratory analyses and revised the manuscript. HD critically revised the manuscript for important intellectual content. TW recruited patients, collected and interpretated data, and critically revised the manuscript for important intellectual content. PR recruited patients and collected data. FM recruited patients, collected and interpretated data, and revised the manuscript for important intellectual content. CN collected data and revised the manuscript. ToD designed the study, collected and interpretated data, and revised the manuscript for important intellectual content. All authors contributed to the article and approved the submitted version.

## Funding

The present study was partly financed by the European Union’s European Regional Development Fund through the INTERREG V program “Alpenrhein-Bodensee-Hochrhein”; Project Number: ABH055. The sponsors had no involvement in the study design, in the collection, analysis, and interpretation of data or the writing of the manuscript or in the decision to submit the manuscript for publication.

## Acknowledgments

The authors sincerely thank all patients participating in this research study and all involved nurses and physicians for supporting patient recruitment and sample collection.

## Conflict of interest

The authors declare that the research was conducted in the absence of any commercial or financial relationships that could be construed as a potential conflict of interest.

## Publisher’s note

All claims expressed in this article are solely those of the authors and do not necessarily represent those of their affiliated organizations, or those of the publisher, the editors and the reviewers. Any product that may be evaluated in this article, or claim that may be made by its manufacturer, is not guaranteed or endorsed by the publisher.
