# Supplementary material for: Circulating syndecan-1 and glypican-4 predict 12-month survival in metastatic colorectal cancer patients
Source: Front Oncol. 2022 Oct 24;12:1045995. doi: 10.3389/fonc.2022.1045995 (PMC9638102; doi:10.3389/fonc.2022.1045995)
Supplement: Supplementary file 1 [file DataSheet_1.pdf]

Supplementary Material

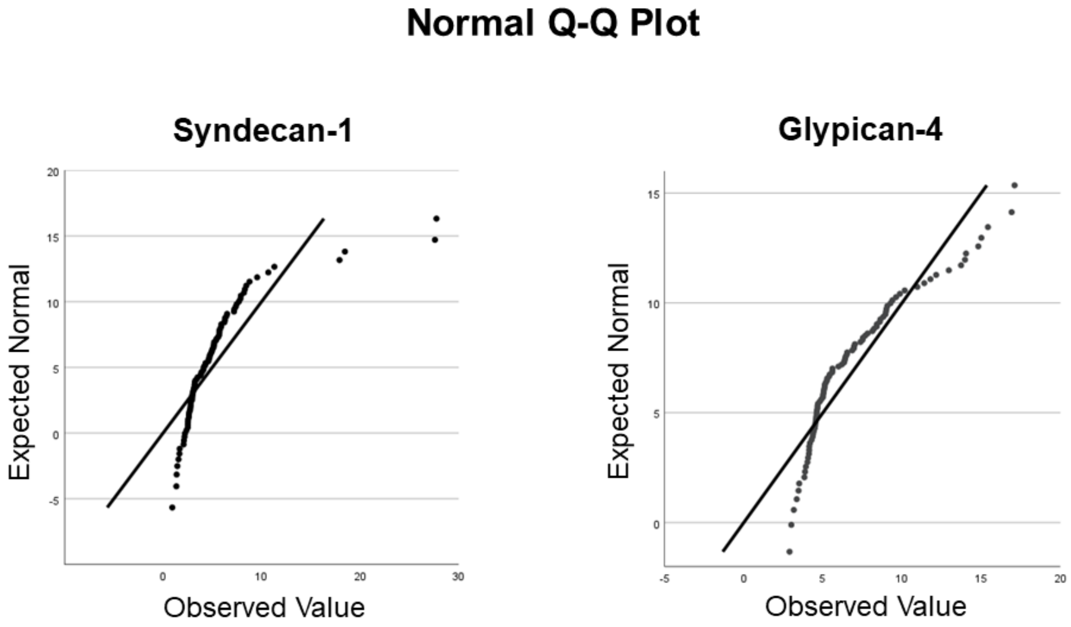

Supplementary Figure 1: Quantile-quantile (Q-Q) plots of SDC1 and GPC4 values show that SDC1 and GPC4 levels were not normally distributed.

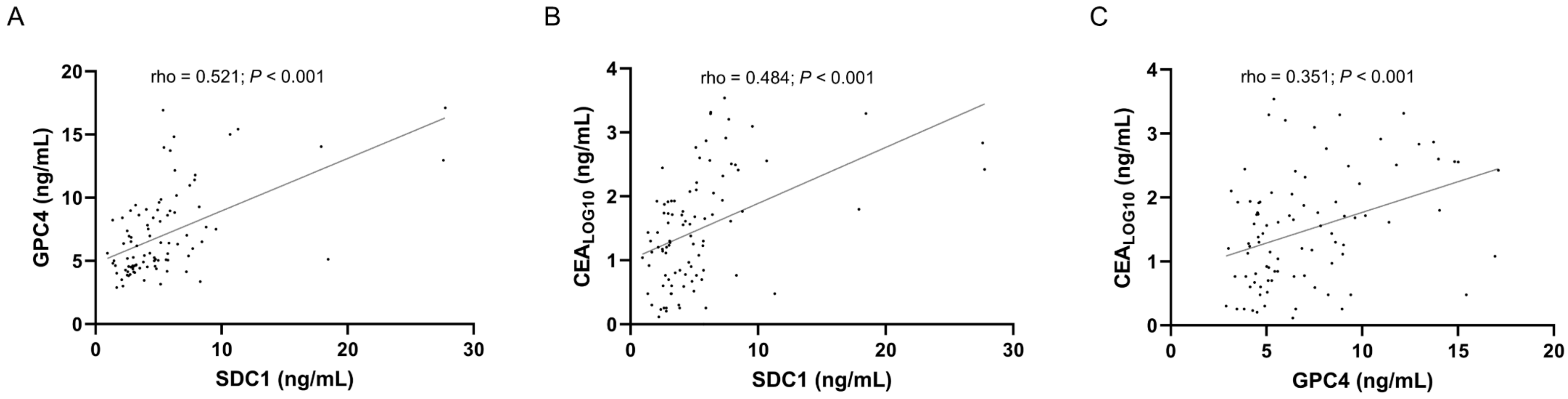

Supplementary Figure 2: Scatter plots visualizing the correlations between a) SDC1 and GPC4, b) SDC1 and CEA, and c) GPC4 and CEA. Due to the broad range of CEA values within the study population, CEA values were log10-transformed for better display. Correlation analyses were performed by calculating non-parametric Spearman rank correlation coefficients (rho). SDC1, syndecan-1; GPC4, glypican-4; CEA, carcinoembryonic antigen.

Supplementary Table 1: Basic clinical and clinicopathological characteristics according to tertiles of syndecan-1

	Tertile 1 (n=31)	Tertile 2 (n=31)	Tertile 3 (n=31)	P_{trend}
**Age, years**	64.4 [54.9-72.1]	63.1 [55.8-73.3]	67.6 [60.7-76.2]	0.270
**Sex**				
Male, N (%)	18 (58.1)	20 (64.5)	23 (74.2)	0.404
Female, N (%)	13 (41.9)	11 (35.5)	8 (25.8)	
**Primary tumor site**				
Colon, N (%)	21 (67.7)	20 (64.5)	21 (67.7)	0.953
Rectum, N (%)	10 (32.3)	11 (35.5)	10 (32.3)	
**Primary tumor sidedness**[†]				
Left, N (%)	22 (78.6)	22 (71.0)	21 (70.0)	0.469
Right, N (%)	6 (21.4)	9 (29.0)	9 (30.0)	
**RAS mutation status**[††]				
Wild-type, N (%)	17 (63.0)	13 (44.8)	15 (53.6)	0.496
Mutated, N (%)	10 (37.0)	16 (55.2)	13 (46.4)	
**Number of metastatic sites**[††]				
1, N (%)	14 (53.8)	18 (62.1)	14 (48.3)	
2, N (%)	10 (38.5)	6 (20.7)	10 (34.5)	0.436
≥3, N (%)	2 (7.7)	5 (17.2)	5 (17.2)	
**Scheduled therapy line**				
1st, N (%)	25 (80.6)	30 (96.8)	29 (93.5)	0.087
2nd, N (%)	6 (19.4)	1 (3.2)	2 (6.5)	

**Scheduled chemotherapy**				
FOLFIRI, N (%)	18 (58.1)	19 (61.3)	18 (58.1)	
FOLFOX, N (%)	7 (22.6)	2 (6.5)	5 (16.1)	0.769
Other chemotherapy, N (%)	6 (19.4)	10 (32.3)	8 (25.8)	
**Scheduled targeted therapy**†††				
No targeted therapy, N (%)	5 (16.1)	5 (17.2)	6 (19.4)	
VEGF-inhibitor therapy, N (%)	16 (51.6)	16 (55.2)	15 (48.4)	0.739
EGFR-inhibitor therapy, N (%)	10 (32.3)	8 (27.6)	10 (32.3)	
**Laboratory parameters**				
CEA, ng/mL	15.0 [4.0-27.2]	18.0 [4.7-58.0]	208.0 [20.0-736]	< 0.001
Syndecan-1, ng/mL	2.55 [2.08-2.82]	4.31 [3.79-4.97]	7.37 [5.92-8.78]	< 0.001
Glypican-4, ng/mL	4.60 [4.01-6.34]	5.61 [4.66-8.63]	8.60 [5.58-12.16]	< 0.001

Missing samples: † n=4; †† n=9; ††† n=2. Differences in baseline characteristics according to syndecan-1 tertiles were tested for statistical significance with Chi-squared tests for trend for categorical and Jonckheere Terpstra tests for continuous variables, respectively. Continuous variables are given as median and interquartile range (defined as the range from the 25th to the 75th percentile).

Supplementary Table 2: Basic clinical and clinicopathological characteristics according to tertiles of glypican-4

	Tertile 1 (n=31)	Tertile 2 (n=31)	Tertile 3 (n=31)	P_{trend}
**Age, years**	61.2 [54.5-71.6]	67.6 [60.2-76.5]	64.9 [57.9-74.3]	0.295
**Sex**				
Male, N (%)	20 (64.5)	20 (64.5)	21 (67.7)	0.953
Female, N (%)	11 (35.5)	11 (35.5)	10 (32.3)	
**Primary tumor site**				
Colon, N (%)	19 (61.3)	20 (64.5)	23 (74.2)	0.533
Rectum, N (%)	12 (38.7)	11 (35.5)	8 (25.8)	
**Primary tumor sidedness**[†]				
Left, N(%)	20 (66.7)	21 (75.0)	24 (77.4)	0.348
Right, N(%)	10 (33.3)	7 (25.0)	7 (22.6)	
**RAS mutation status**[††]				
Wild-type, N (%)	18 (64.3)	11 (39.3)	16 (57.1)	0.594
Mutated, N (%)	10 (35.7)	17 (60.7)	12 (42.9)	
**Number of metastatic sites**[††]				
1, N (%)	17 (60.7)	13 (48.1)	16 (55.2)	
2, N (%)	9 (32.1)	9 (33.3)	8 (27.6)	0.424
≥3, N (%)	2 (7.1)	5 (18.5)	5 (17.2)	
**Scheduled therapy line**				
1st, N (%)	28 (90.3)	27 (87.1)	29 (93.5)	0.691
2nd, N (%)	3 (9.7)	4 (12.9)	2 (6.5)	

**Scheduled chemotherapy**				
FOLFIRI, N (%)	18 (58.1)	15 (48.4)	22 (71.0)	
FOLFOX, N (%)	5 (16.1)	8 (25.8)	1 (3.2)	0.557
Other chemotherapy, N (%)	8 (25.8)	8 (25.8)	8 (25.8)	
**Scheduled targeted therapy**†††				
No targeted therapy, N (%)	8 (26.7)	4 (13.3)	4 (12.9)	
VEGF-inhibitor therapy, N (%)	12 (40.0)	19 (63.3)	16 (51.6)	0.199
EGFR-inhibitor therapy, N (%)	10 (33.3)	7 (23.3)	11 (35.5)	
**Laboratory parameters**				
CEA, ng/mL	17.2 [4.0-54.9]	14.0 [5.0-113.0]	58.0 [18.0-365.0]	0.005
Syndecan-1, ng/mL	2.96 [2.51-3.86]	4.65 [2.82-5.83]	5.91 [4.58-7.90]	< 0.001
Glypican-4, ng/mL	4.28 [3.87-4.57]	5.61 [5.12-6.53]	9.62 [8.63-13.73]	< 0.001

Missing samples: † n=4; †† n=9; ††† n=2. Differences in baseline characteristics according to glypican-4 tertiles were tested for statistical significance with Chi-squared tests for trend for categorical and Jonckheere Terpstra tests for continuous variables, respectively. Continuous variables are given as median and interquartile range (defined as the range from the 25th to the 75th percentile).
